# Reviewer acknowledgement 2013

**DOI:** 10.1186/2052-0492-2-4

**Published:** 2014-02-03

**Authors:** Satoshi Gando

**Affiliations:** Division of Acute and Critical Care Medicine, Department of Anesthesiology and Critical Care Medicine, Hokkaido University Graduate School of Medicine, Hokkaido, Japan

## Abstract

The editors of *Journal of Intensive Care* would like to thank all our reviewers who have contributed to the journal in Volume 1 (2013).

**Toshikazu Abe**

Japan

**Fumimasa Amaya**

Japan

**Kohkichi Andoh**

Japan

**Daniele Bollero**

Italy

**Moritoki Egi**

Japan

**Yuji Fujino**

Japan

**Seitaro Fujishima**

Japan

**Satoshi Fujita**

Japan

**Shigeki Fujitani**

Japan

**Satoru Hanada**

Japan

**Satoru Hashimoto**

Japan

**Mineji Hayakawa**

Japan

**Yoshiro Hayashi**

Australia

**Hirofumi Hino**

Japan

**Toshiaki Ikeda**

Japan

**Hideaki Imanaka**

Japan

**Hideo Inaba**

Japan

**Hironori Ishihara**

Japan

**Eiji Isotani**

Japan

**Takashi Ito**

Japan

**Gavin Joynt**

Hong Kong

**Motoshi Kainuma**

Japan

**Yasuyuki Kakihana**

Japan

**Masato Kato**

Japan

**Nobuyuki Katori**

Japan

**Atsushi Kawaguchi**

Canada

**Kaneyuki Kawamae**

Japan

**Tatsuya Kawasaki**

Japan

**Kosaku Kinoshita**

Japan

**Yutaka Kondo**

Japan

**Yoshifumi Kotake**

Japan

**Joji Kotani**

Japan

**Toru Kotani**

Japan

**Yasuhiro Kuroda**

Japan

**Jeffrey Lipman**

Australia

**John Marshall**

Canada

**Naoyuki Matsuda**

Japan

**Hikoro Matsui**

Japan

**Chieko Mitaka**

Japan

**Hiroshi Morimatsu**

Japan

**Yuji Morimoto**

Japan

**Hiroshi Morisaki**

Japan

**Kiyoshi Moriyama**

Japan

**Takaya Morooka**

Japan

**Satoshi Nakagawa**

Japan

**Susumu Nakagawa**

Japan

**Masaki Nakane**

Japan

**Minoru Nakano**

Japan

**Koichi Nakao**

Japan

**Hiromichi Narumiya**

Japan

**Shinichi Nishi**

Japan

**Hiroshi Nishida**

Japan

**Masaji Nishimura**

Japan

**Takeshi Nomura**

Japan

**Hiroshi Nonogi**

Japan

**Shin Nunomiya**

Japan

**Shigeto Oda**

Japan

**Kazufumi Okamoto**

Japan

**Mutsuo Onodera**

Japan

**Kiyohiro Oshima**

Japan

**Luc Puis**

Belgium

**Yoshiro Sakaguchi**

Japan

**Masamitsu Sanui**

Japan

**Nobuo Sasaki**

Japan

**Nobuaki Shime**

Japan

**Yoshito Shiraishi**

Japan

**Kazuya Sobue**

Japan

**Naosuke Sugai**

Japan

**Takeshi Suzuki**

Japan

**Yoshio Tahara**

Japan

**Shinhiro Takeda**

Japan

**Tetsuhiro Takei**

Japan

**Naoshi Takeyama**

Japan

**Ryosuke Tsurura**

Japan

**Osamu Umegaki**

Japan

**Yutaka Usuda**

Japan

**Masahiko Uzura**

Japan

**Hiroyuki Watanabe**

Japan

**Hiroshi Yamada**

Japan

**Takeshi Yamamoto**

Japan

**Hidekazu Yukioka**

Japan

